# Synovial myxoma or myxosarcoma? Lymph node metastasis in 2 dogs

**DOI:** 10.1177/10406387241257254

**Published:** 2024-06-03

**Authors:** Imaine Glahn, Taryn A. Donovan, Christof A. Bertram

**Affiliations:** University of Veterinary Medicine Vienna, Vienna, Austria; The Schwarzman Animal Medical Center, New York, NY, USA; University of Veterinary Medicine Vienna, Vienna, Austria

**Keywords:** dogs, lymphovascular invasion, metastasis, pathology, synovial myxoma

## Abstract

Synovial myxoma, a rare joint tumor in dogs, has traditionally been considered benign, acknowledging that local invasion into regional tissues including bone may be present. Given the diagnostic challenges in distinguishing synovial myxoma from other joint lesions through clinical features and diagnostic imaging, definitive diagnosis relies on characteristic gross and histologic features. Within the inner surface of the joint capsule, synovial myxomas form nodules of stellate-to-spindle cells within abundant myxomatous matrix. We present here 2 cases of synovial myxoma with metastasis to regional lymph nodes and compare these 2 cases to 3 cases without evidence of lymph node metastasis. Aside from lymphovascular invasion in one case with metastasis, there were no overt histologic features of the primary tumor to suggest aggressive biologic behavior. The finding of lymph node metastasis warrants reconsideration of the term “synovial myxoma” for this neoplasm. We suggest the term “synovial myxosarcoma,” considering that histologic features of the primary tumor do not predict biologic behavior. Our case series highlights the importance of lymph node sampling in suspected synovial myxosarcoma cases as well as thorough histologic examination, emphasizing careful evaluation for lymphovascular invasion.

Synovial myxoma is considered the second most common joint tumor in dogs, following histiocytic sarcoma, although it is exceptionally rare, with only 52 reported cases to date.^1,3,4,8,11^ Most commonly affecting middle-aged dogs (median age: 8.5 y) of large breeds, synovial myxoma typically occurs within the stifle joint, although occurrences in the carpus, digits, and tarsus have been described. Diagnosis often follows prolonged lameness over weeks to years.^1,4,6^ Differentiating between synovial myxoma and other joint lesions through clinical diagnosis and diagnostic imaging poses a challenge due to similar presenting clinical and radiographic findings^4,7^; hence, the prognosis can be significantly variable.^3,7,11,12^ Ultimately, synovial myxomas are diagnosed based on their characteristic histomorphologic appearance, enabling differentiation from other reported joint lesions in dogs.^3,5,11^

Despite its documented favorable prognosis, with a recurrence rate of only 10%, and its overall benign behavior, instances of invasion into bone and surrounding tissues, as well as infiltration along fascial planes, have been reported.^3,4,8^ None of the 52 cases of canine synovial myxoma reported have displayed lymph node or distant metastasis, which had justified the benign classification of this tumor entity.^1,3,4,8,11^ We present here a series of 5 cases of tumors originally classified as synovial myxoma in dogs, 2 of which had metastasis to local lymph nodes.

Cases were identified from diagnostic archives of 2 veterinary pathology institutions: The Schwarzman Animal Medical Center (New York, NY, USA; *n* = 4) and the University of Veterinary Medicine Vienna (Vienna, Austria; *n* = 1). Surgical biopsy reports were analyzed for demographic information, tumor location, and clinical history. Cases without tissue samples of regional lymph nodes were excluded. Archived tissue blocks were retrieved, and routine H&E-stained sections were produced for re-evaluation of tumor morphology and assessment of histologic criteria of malignancy (Table 1). Histologic sections of popliteal (*n* = 4, primary tumors of the stifle and tarsus) and prescapular (*n* = 1, primary tumor of the carpus) lymph nodes for all 5 cases were examined by one American College of Veterinary Pathologists–certified pathologist (CA Bertram) to determine the presence of metastasis. Lymphovascular invasion (LVI, according to the Veterinary Cancer Guidelines and Protocols definitions)^9^ and invasion into surrounding tissue were evaluated as absent or present. For better evaluation of LVI, primary tumors with lymph node metastasis were immunolabeled with platelet endothelial cell adhesion molecule 1 (PECAM1, E4, CD31) monoclonal mouse antibodies (Santa Cruz Biotechnology) at a dilution of 1:750 after pretreatment with citric acid (pH 6) to highlight endothelium of blood or lymph vessels.^14^ Nuclear pleomorphism was classified as mild = uniform and regular nuclei with minimal anisokaryosis; moderate = increased variability in size (<2-fold difference) and shape; or severe = nuclei varying >2-fold in size.

**Table 1.** Demographic characteristics, primary tumor site, lymph node metastasis, lymph node site, and histologic malignancy criteria of the 5 dogs with synovial myxosarcoma.

Case	Breed	Age, y	Primary tumor site	LNM	Lymph node site	LVI	Bone invasion	MC	Nuclear pleomorphism	Cellular density
1	Labrador	9	Tarsus	Yes	L popliteal	Yes	Yes	1	Moderate	Mild
2	Mixed, 18.5 kg	9	Stifle	Yes	L popliteal	No	Yes	1	Mild	Mild
3	Golden Retriever	8	Carpus	No	R prescapular	No	Yes	9	Moderate	Moderate
4	Shepherd mix	12	Stifle	No	L popliteal	No	No	0	Mild	Mild
5	Rottweiler	9	Stifle	No	R popliteal	No	No	0	Moderate	Mild

L = left; LNM = lymph node metastasis; LVI = lymphovascular invasion; MC = mitotic count per 2.37 mm^2^; R = right.

We included 5 patients with synovial myxomatous tumors in our study (Table 1). The group consisted of 3 spayed females and 2 castrated males, with a median age of 9.4 y (range: 8–12 y). The most common location was the stifle joint (*n* = 3), with one case each occurring in the tarsus and carpus.

Macroscopically, the neoplastic proliferations manifested as numerous soft, pale nodules and pockets filled with viscous fluid. The nodules covered a large portion of the inner surface of the joint capsule, occasionally extending beyond the capsule (*n* = 3) and secreting viscous fluid upon incision (Fig. 1A). Invasion into the adjacent bone was documented in 3 cases, 2 of which had lymph node metastasis.

**Figure 1.**
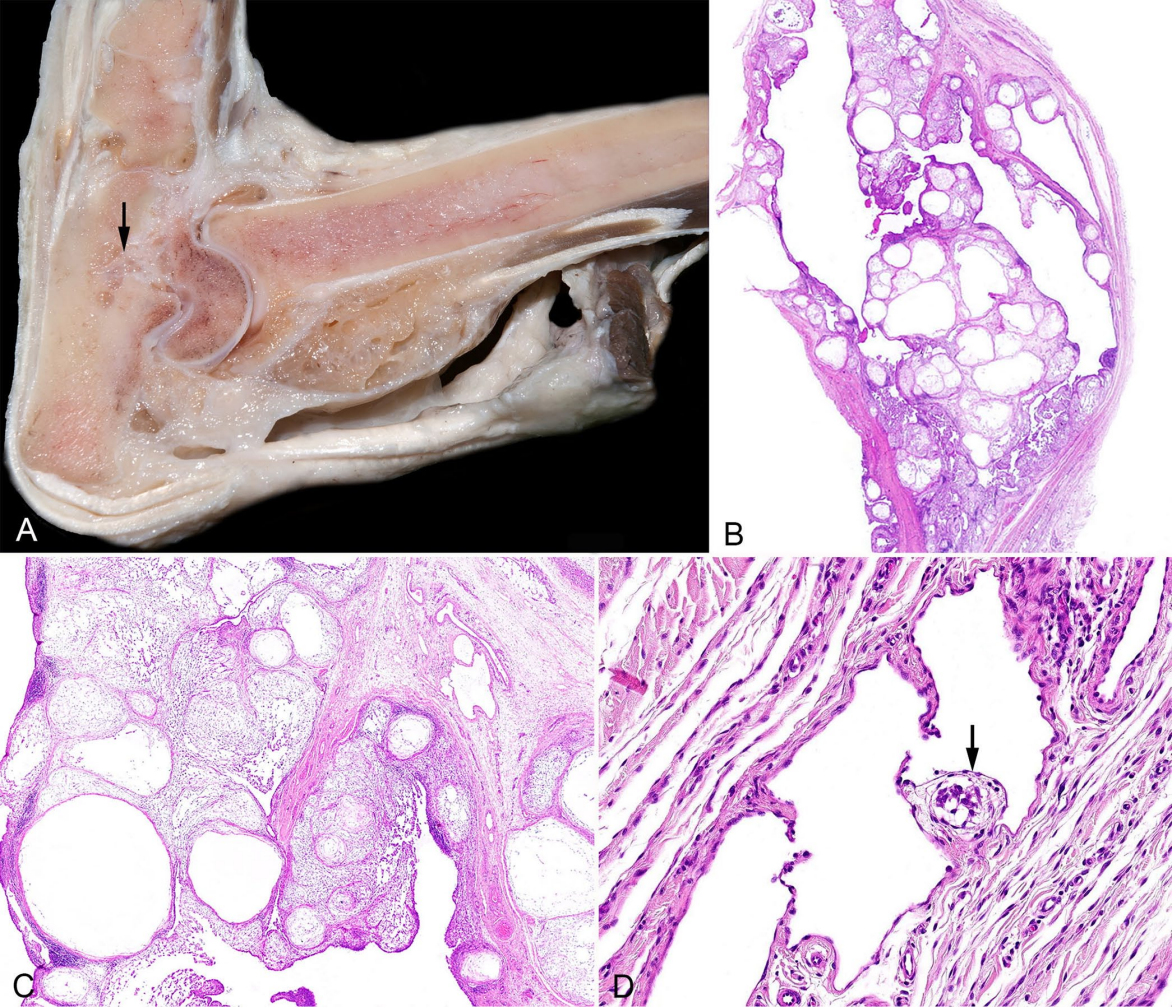
Gross and histologic images of the synovial myxosarcoma and regional lymph node from case 1. **A.** Cross-section through the primary tumor of the left tarsal joint with invasion into the calcaneus (arrow). **B.** Subgross histologic section of the primary tumor. H&E. **C.** Higher magnification of image B. **D.** Extra-tumoral vessel (consistent with dilated lymph vessel) adjacent to the primary tumor with neoplastic embolus attached to a valve (arrow) and potentially covered by endothelium. H&E.

Histologically, these tumors were characterized by multilobular nodules of various sizes composed of sparsely dispersed stellate-to-spindle cells surrounded by abundant myxoid matrix and separated by collagenous connective tissue (Fig. 1B, 1C; Suppl. Fig. 1A, 1B). Neoplastic cells were elongate-to-oval, with round-to-oval nuclei, and moderate amounts of amphophilic cytoplasm. Macroscopic and histologic findings were consistent with synovial myxoma in all 5 cases (Table 1). Cellular density was low in all tumors (as is characteristic of this type of tumor), except for one case that contained some areas with higher cellular density and sparse myxoid stroma. Reactive changes of lymph nodes, such as follicular and paracortical hyperplasia, edema, hemosiderosis and erythrophagocytosis, were observed in all sampled lymph nodes. In cases 1 and 2, lymph nodes (with histologic diameters of 17 and 10 mm, respectively) contained neoplastic populations similar to those observed in the primary tumor (Fig. 2A–C; Suppl. Fig. 1C), featuring a myxomatous matrix (confirmed by alcian blue stain, Fig. 2B–D; Suppl. Fig. 1D) and islands of spindle-to-stellate cells, consistent with “synovial myxoma metastasis.” In both cases, the metastatic foci (of up to 0.7 and 1.5 mm diameter) were located throughout the entire sinus system, mostly in the subcapsular sinus but also extending into the paratrabecular and medullary sinus.

**Figure 2.**
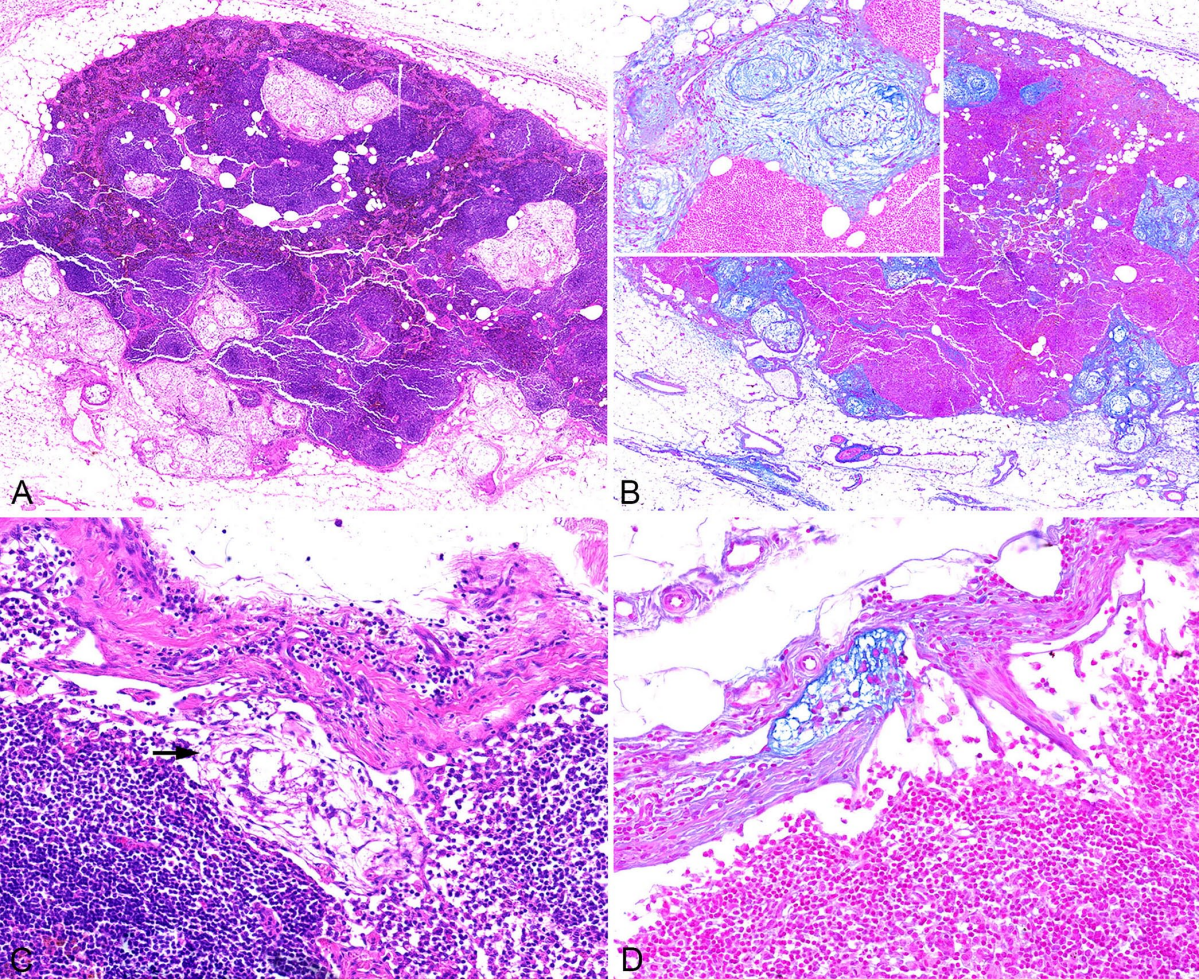
Histologic images of the regional lymph nodes from cases 1 and 2 of synovial myxosarcoma. **A.** Lymph node section from case 2 with metastases in the sinus system, most notably in the subcapsular sinus. H&E. **B.** Consecutive section of the lymph node in image A highlighting the production of mucinous extracellular matrix by the tumor cells. Inset: higher magnification with metastatic foci in the subcapsular sinus. Alcian blue. **C.** Lymph node section of case 1 with metastasis in subcapsular sinus (arrow). H&E. **D.** Consecutive lymph node section with metastasis in a lymph vessel extending into the subcapsular sinus. Alcian blue.

Notably, the H&E image of case 1 had a neoplastic cell cluster (embolus) within the wall of a dilated peritumoral vessel, which contained valves but no muscle, and very few red blood cells, indicative of a lymph vessel (Fig. 1D). The neoplastic embolus was attached to a vascular valve; however, it is unclear whether it was covered by endothelium. This observation strongly suggests the occurrence of LVI, supporting the hypothesis that the tumor may have invaded an intratumoral lymph vessel with subsequent spread to an extratumoral location and stoppage by the valve. Immunohistochemistry (IHC) with antibodies against PECAM1 confirmed a vascular space with endothelial lining, but the tumor mass was not present in this consecutive section, thus endothelial coverage cannot be confirmed.

Lymph node metastasis was not correlated with increased histomorphologic criteria of malignancy in the primary tumor (i.e., cellular and nuclear pleomorphism); however, identification of LVI (considered a criterion of malignancy) was found in one case with lymph node metastasis (Table 1). Patient follow-up of cases with lymph node metastases was requested through the submitting clinician or the owner. Case 1 was still alive 13 mo after surgery and without evidence of further tumor spread in clinical staging. Case 2 was treated with chemotherapy (unknown drug) and died 8 mo after surgery, with an unknown cause of death.

Our findings provide proof of regional lymph node metastasis and LVI in canine “synovial myxoma,” which necessitates revision of the current terminology and recognition of a malignant form of this tumor type. Based on an extensive review of the literature, we were able to locate only one previous documentation of a metastatic synovial myxomatous tumor presented at a slide conference^2^; however, images of the affected lymph nodes are not available. Notably, this case, despite the description of metastasis, was diagnosed as a “synovial myxoma” emphasizing the lack of clarity regarding the terminology of this tumor entity.^2^ The presence of lymph node metastases justifies the diagnosis of “synovial myxosarcoma,” considering that metastasis is the defining criterion of malignancy.

In a 2020 book, a distinction was suggested between synovial myxoma and myxosarcoma, acknowledging the lack of clear criteria to do so.^13^ Our small case series has shown the inability to identify the metastatic cases based on traditional criteria of histologic malignancy of the primary tumor. Thus, we suggest the term “synovial myxosarcoma” for all cases, with the understanding that metastasis is uncommon based upon the literature. This interpretation of malignancy is also consistent with the tendency of the neoplasm for local invasive growth into surrounding soft tissue and bone. It is unknown whether regional lymph node metastases portend additional aggressive behavior (i.e., metastasis to other lymph nodes and distant organs); however, pathologists should be aware that these neoplasms can metastasize to local lymph nodes. We encourage oncologists and surgeons to sample local lymph nodes when this tumor type is suspected, which will allow for more accurate classification of this tumor.

Lymph node metastases in our 2 cases of canine synovial myxosarcoma were not associated with histologic criteria of malignancy of the primary tumor, suggesting that the biological behavior of these neoplasms may be difficult to predict histologically. The exceptional finding might be LVI, which was present in 1 of 2 synovial myxosarcomas with lymph node metastasis. LVI is generally used as a prognostic marker to indicate more aggressive biologic behavior and an increased probability of lymph node metastasis in both human and animal tumors.^10^ To evaluate whether LVI may be a reliable (sensitive and specific) marker for metastasis in canine synovial myxosarcoma, we recommend that intra- and extra-tumoral screening of lymph and blood vessels be incorporated as part of the diagnostic workflow. In addition, types and number of vessels with LVI should be recorded. Robust criteria needed to confirm LVI include adhesion of thrombotic material to the neoplastic embolus and/or documented infiltration of the vessel wall.^9,10^ IHC for endothelial cells could be helpful for verifying the presence of tumor cells within the lumen of blood and/or lymph vessels, thereby aiding differentiation from pseudo-LVI (i.e., stromal retraction artifact),^10^ as has been shown for canine mammary tumors.^15^ In our case, LVI was interpreted based on a neoplastic embolus attached to a vascular valve. It remains unclear whether the embolus was covered by endothelium as the consecutive section for IHC did not include the small embolus. However, considering that the affected valve was 2 mm away from the tumor, we suspect intra-tumoral LVI with spread to this extra-tumoral vessel and entrapment by the valve.

Our retrospective case series included only 5 cases, which does not allow conclusions to be drawn about the frequency of lymph node metastasis, thus precluding identification of the biologic relevance to patient outcome and therapeutic success. Further validation with a larger study group is required to confirm our findings. It is noteworthy that our study only included excisional biopsies of regional lymph nodes, with only one or a few cross-sections per node. Retrospectively, it cannot be determined if the sampled lymph nodes were tributary, or if metastasis had occurred beyond the lymph nodes. The identification and evaluation of lymph nodes through diagnostic imaging, as conducted previously,^12^ may not be sufficient to identify small metastatic foci without marked lymph node enlargement, as observed in our study, and thus may not be able to differentiate aggressive behavior. Therefore, it is crucial to perform routine lymph node sampling and thoroughly evaluate both the primary tumor and lymph nodes, with emphasis on careful evaluation of malignancy criteria and LVI to identify cases with metastasis.

## Supplemental Material

sj-pdf-1-vdi-10.1177_10406387241257254 – Supplemental material for Synovial myxoma or myxosarcoma? Lymph node metastasis in 2 dogs Supplemental material, sj-pdf-1-vdi-10.1177_10406387241257254 for Synovial myxoma or myxosarcoma? Lymph node metastasis in 2 dogs by Imaine Glahn, Taryn A. Donovan and Christof A. Bertram in Journal of Veterinary Diagnostic Investigation
