# Supplementary material for: Synovial myxoma or myxosarcoma? Lymph node metastasis in 2 dogs
Source: J Vet Diagn Invest. 2024 Jun 3;36(6):874–8. doi: 10.1177/10406387241257254 (PMC11514113; doi:10.1177/10406387241257254)
Supplement: sj-pdf-1-vdi-10.1177_10406387241257254 – Supplemental material for Synovial myxoma or myxosarcoma? Lymph node metastasis in 2 dogs [file sj-pdf-1-vdi-10.1177_10406387241257254.pdf]

JVDI: Supplemental material

Glahn I, et al. Synovial myxoma or myxosarcoma? Lymph node metastasis in 2 dogs

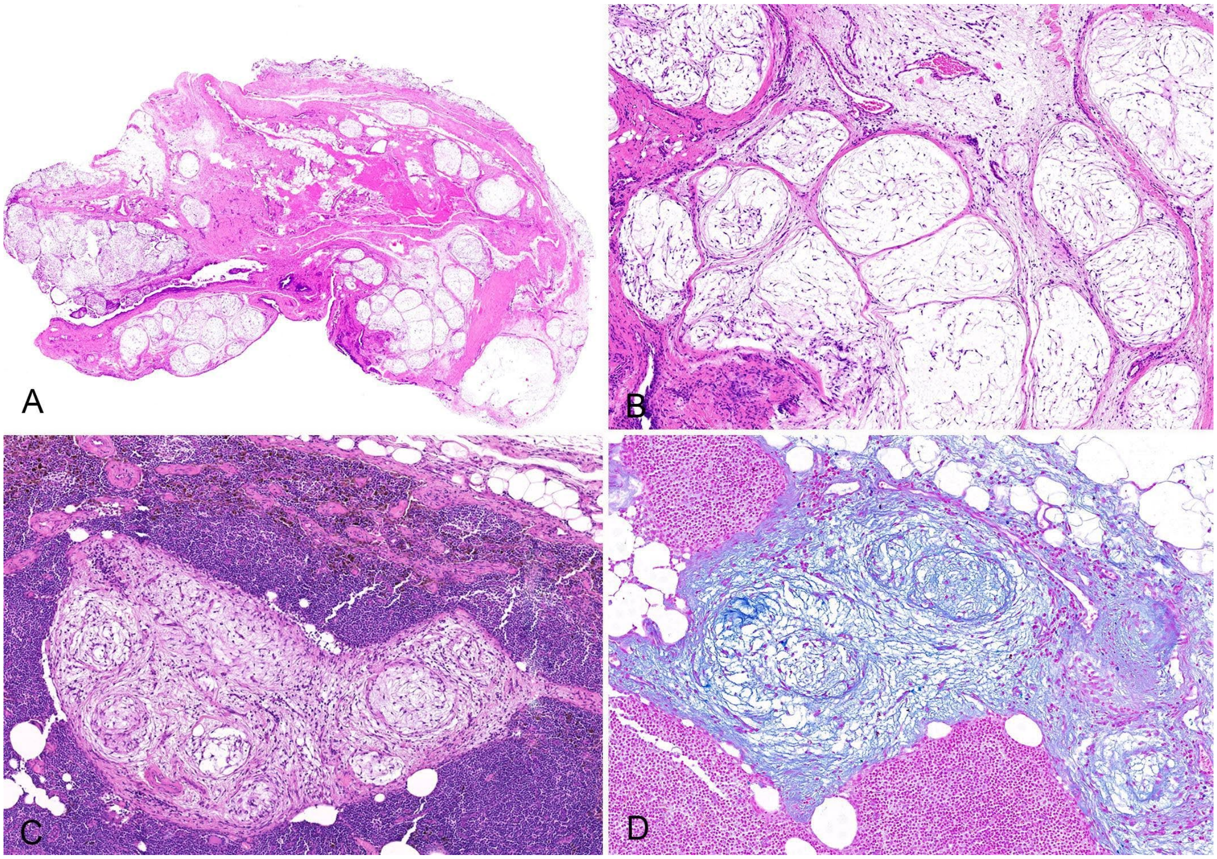


**Supplemental Figure 1.** Histologic images of the synovial myxosarcoma and regional lymph node from case 2. **A.** Subgross image of the primary tumor of the left stifle joint. H&E. **B.** Higher magnification of image A. **C.** Regional lymph node with metastasis in the cortex. H&E. **D.** Consecutive section of image C. Alcian blue.
